# NSCR-Based DenseNet for Lung Tumor Recognition Using Chest CT Image

**DOI:** 10.1155/2020/6636321

**Published:** 2020-12-16

**Authors:** Zhou Tao, Huo Bingqiang, Lu Huiling, Yang Zaoli, Shi Hongbin

**Affiliations:** ^1^School of Computer Science and Engineering, North Minzu University, Yinchuan 750021, China; ^2^School of Science, Ningxia Medical University, Yinchuan 750004, China; ^3^College of Economics and Management, Beijing University of Technology, Beijing 100124, China; ^4^Urinary Surgery, General Hospital of Ningxia Medical University, Yinchuan 750004, China

## Abstract

Nonnegative sparse representation has become a popular methodology in medical analysis and diagnosis in recent years. In order to resolve network degradation, higher dimensionality in feature extraction, data redundancy, and other issues faced when medical images parameters are trained using convolutional neural networks. Lung tumors in chest CT image based on nonnegative, sparse, and collaborative representation classification of DenseNet (DenseNet-NSCR) are proposed by this paper: firstly, initialization parameters of pretrained DenseNet model using transfer learning; secondly, training DenseNet using CT images to extract feature vectors for the full connectivity layer; thirdly, a nonnegative, sparse, and collaborative representation (NSCR) is used to represent the feature vector and solve the coding coefficient matrix; fourthly, the residual similarity is used for classification. The experimental results show that the DenseNet-NSCR classification is better than the other models, and the various evaluation indexes such as specificity and sensitivity are also high, and the method has better robustness and generalization ability through comparison experiment using AlexNet, GoogleNet, and DenseNet-201 models.

## 1. Introduction

Chest CT images offer the advantages of easy access, cost-effectiveness, and low radiation dosage needed, making it the most common screening procedure in daily clinical practice. Diagnostic testing of multiple diseases of the chest from CT images by radiologists can provide useful references for the diagnosis and treatment of lung diseases. Lung cancer [1] is one of the malignant tumors with a high rate of morbidity and mortality, posing a serious threat to human health. Early diagnosis and early detection are crucial to the treatment of lung cancer. Computer-assisted diagnostic technology (CAD) [2] has been widely used in the diagnosis and treatment of various diseases, especially lung cancer detection, which is one of the most common applications of CAD technology. The introduction of computer-aided diagnosis technology has an important and positive effect on the early detection and diagnosis of lung cancer, so it has great prospects for development in the field of assisting doctors in diagnosing and treating lung cancer.

In recent years, deep learning [3] had achieved great success in the field of image processing due to its excellent learning capabilities. Deep learning, exemplified by DenseNet [4], has been increasingly applied in the field of medical imaging; good results have been achieved in clinically assisted classification, identification, detection, and segmentation for benign and malignant tumors, brain functions, cardiovascular diseases, and other major diseases. Residual neural networks (ResNet) [5, 6] reduce feature redundancy and reuse existing features by sharing parameter shortcut connections and preserving intermediate features. Khened et al. [7] proposed a fully convolutional multiscale residual DenseNets for cardiac segmentation and automated cardiac diagnosis using ensemble of classifiers. In Alzheimer's disease diagnosis, hippocampus analysis by combination of 3-D DenseNet and shapes are putted forward by Cui and Liu [8]. Tong et al. [9] proposed a channel-attention-based DenseNet network for remote sensing image scene classification. However, the trade-off is that it is difficult to rediscover the underlying features using high-level information; DenseNet effectively leverages high-level information to rediscover new features at the bottom layer, enhancing feature transmission across the network and enabling enhanced feature reuse, effectively reducing the number of parameters. Chen et al. [10] proposed a new DenseNet and ResNet-based dual asymmetric feature learning network, DualCheXNet, which uses two homogeneous DCNNs to learn each other supplemented with more accurate features for multilabeled thoracic disease classification, which is relatively robust; Dai et al. [11] proposed the improved lung nodule classification identification algorithm based on DenseNet; the model is based on DenseNet and uses intermediate dense projection method to obtain three-dimensional information about pulmonary nodules and train the network using Focal Loss to enable the network to focus on learning the difficult resolved lung nodules, with good experimental results; Zhu and Qin [12] proposed an improved U-Net convolutional neural network lung nodule detection algorithm using convolutional and pooling operation to retrieve high-level features, enable high-speed flow of feature information between input and output layers through DenseNet, and generate in combination with expansion convolution. Multiscale features improve the utilization of low-level features of pulmonary nodules. Li et al. [13] used a DenseNet for computer-aided diagnosis of lung cancer, which uses a patch-based, multiresolution DenseNet to extract features and classify them using four different integration methods.

Sparse representation (SR) and collaborative representation (CR) have become a popular methodology in pattern classification and computer vision for computer-aided diagnosis (CAD) and tumor recognition in recent years [14]. These methods first encode the query sample as a linear combination of the given training samples and then assign the query sample to the corresponding class with the minimal distance or approximation error. One seminal work in this category is the sparse representation- (SR-) based classifier (SRC). Sparse representation models often contain two stages: sparse coding and dictionary learning. Li et al. [15] propose a nonnegative dictionary-based sparse representation and classification scheme for ear recognition. The nonnegative dictionary includes the Gabor feature dictionary extracted from the ear images and nonnegative occlusion dictionary learned from the identity occlusion dictionary. A test sample with occlusion can be sparsely represented over the Gabor feature dictionary and the occlusion dictionary. The sparse coding coefficients are noted with nonnegativity and much more sparsity, and the nonnegative dictionary has shown increasing discrimination ability. Mi et al. [16] propose a robust supervised sparse representation (RSSR) model, which uses a two-phase robust representation to compute a sparse coding vector. Huber loss is employed as the fidelity term in the linear representation, which improves the competitiveness of correct class in the first phase. Then, training samples with weak competitiveness are removed by supervised way. In the second phase, the competitiveness of correct class is further boosted by Huber loss. Zhang et al. [17] propose a nonlinear nonnegative sparse representation model: NNK-KSVD. In the sparse coding stage, a nonlinear update rule is proposed to obtain the sparse matrix. In the dictionary learning stage, the proposed model extends the kernel KSVD by embedding the nonnegative sparse coding. The proposed nonnegative kernel sparse representation model was evaluated on several public image datasets for the task of classification. Fuzzy discriminative sparse representation (FDSR) is proposed by Ghasemi et al. [18]; the proposed fuzzy terms increase the interclass representation difference and the intraclass representation similarity. Also, an adaptive fuzzy dictionary learning approach is used to learn dictionary atoms. A robust sparse representation for medical image classification is proposed based on the adaptive type-2 fuzzy learning (T2-FDL) system by Ghasemi et al. [19]. In the proposed method, sparse coding and dictionary learning processes are executed iteratively until a near-optimal dictionary is obtained. Moradi and Mahdavi-Amiri [20] propose a sparse representation-based method for segmentation and classification of lesion images. The main idea of our framework is based on a kernel sparse representation, which produces discriminative sparse codes to represent features in a high-dimensional feature space. Our novel formulation for discriminative kernel sparse coding jointly learns a kernel-based dictionary and a linear classifier. We also present an adaptive K-SVD algorithm for kernel dictionary and classifier learning. In order to solve the semantic gap problem between low-level features and high-level image semantic, which will largely degrade the classification performance, Zhang et al. [21] propose a multiscale nonnegative sparse coding-based medical image classification algorithm.

This paper presents methods for classification of for benign and malignant lung tumors based on non-negative, sparse, and collaborative representation classification of DenseNet (DenseNet-NSCR). First, CT modal medical images were collected and preprocessed. The dataset is then trained in a DenseNet to construct a DenseNet model to extract the full connection layer feature vector. It was concluded with the results of lung tumor classification in the NSCR classifier, compared by a total of nine models, AlexNet+SVM, AlexNet+SRC, AlexNet+NSCR, GoogleNet+SVM, GoogleNet+SRC, GoogleNet+NSCR, DenseNet+SVM, DenseNet+SRC, and DenseNet+NSCR. The DenseNet+NSCR model outperforms the other models with better robustness and generalization capabilities.

## 2. Basic Principle

### 2.1. The Basic Structure of DenseNet

**Figure 1:**
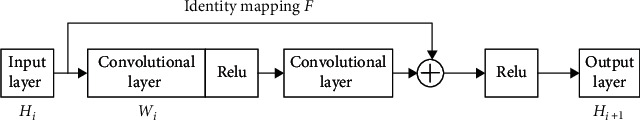
Residual block.

DenseNet is typically composed of multiple Dense Blocks and transition layer structures overlap to form a multilayer neural network. Its internal Dense Block structure uses the residual neural network's shortcut connection [5] structure. The deep residual neural network is usually composed of multiple residual block structures overlapping each other. Neighboring convolutional layers are connected by a shortcut to form a residual block. The residual block structure is shown in Figure 1, where *H*_*i*_ is input, *H*_*i*+1_ is output, *W*_*i*_ is weight, and *F* denotes the identity mapping. The residual block mapping is represented in Figure 1 as

(1) Hi+1=ReluHi+FHi,Wi.

**Figure 2:**
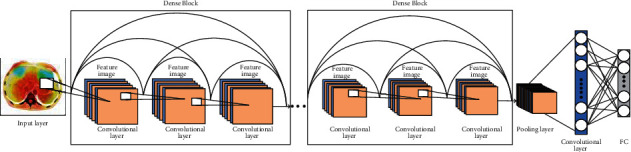
Residual neural network, resNet-101 diagram structure.

The DenseNet structure uses dense connections in model building as shown in Figure 1, where the current network layer is connected to each subsequent layer. The feature map within each Dense Block is of the same size, and the features learned by the DenseNet are reused within the network. The dense connections between the DenseNet layers facilitate the flow of information throughout the network. Its nonlinear function is shown in Eq. (2) where *x*_*i*_ denotes the output of layer l. [*x*_0_  *x*_1_  *x*_2_ ⋯ *x*_*l*−1_] indicates the collocation of feature maps from the input layer to the l-1 layer. *H*_*i*_ denotes the nonlinear function which is a combined operation containing the batch normalization (BN) layer, the Relu layer, and the convolutional layer. As a result, the training of the deep network becomes more efficient and the performance of the model is improved as shown in Figure 2.

DenseNet has fewer parameters for network training compared to ResNet networks. Also, the use of dense connections alleviates the overfitting problem for models with small datasets. For the transition layer, it mainly connects two Dense Blocks, which contain a 1 × 1 convolution and 2 × 2 average pooling to reduce the feature map size. If the Dense Block of the previous layer outputs *m* feature maps, the transition layer can generate *θ* feature maps, where 0 ≤ *θ* ≤ 1 is called the compression factor; when *θ* = 1, the feature map remains unchanged; when *θ* < 1, the transition layer can further compress the model. In this paper, DenseNet's *k* = 32 and *θ* = 0.5 are used.

(2) $$\begin{array}{c} x_{l}=H_{i}\left(\left[x_{0}x_{1}x_{2}\cdots x_{l-1}\right]\right). \end{array}$$

DenseNet has the following features: firstly, DenseNet effectively alleviates the gradient vanishing problem caused by an overly deep network. DenseNet effectively strengthens feature forward transmission by acquiring the loss function of all preceding layers for each layer, so that deeper networks can be trained; secondly, compared to ResNet, which uses summation to transmission features, DenseNet uses inception's concatenation channel merge, which merges all previous layer outputs together as the current input, thus significantly improving feature transmission efficiency; thirdly, residual neural networks reduce feature redundancy and reuse existing features by sharing parameters across layers and preserving intermediate features, with the disadvantage that it is difficult to rediscover the underlying features using high-level information; DenseNet effectively leverages high-level information to rediscover new features at the bottom layer, enhancing feature transmission across the network and enabling and enhancing feature reuse; fourthly, DenseNet effectively reduces the number of parameters compared to ResNet which has a larger number of parameters.

### 2.2. NSCR Algorithm

There are many redundant or irrelevant features in high-dimensional data, thus facing the curse of dimensionality. On one hand, high computational time and space are required; on the other hand, problems such as overfitting occur in classification tasks. Therefore, data dimension reduction is a challenging task in machine learning. The sparse representation of high-dimensional feature data is one of the recent research hotspots in the field of machine learning, and SRC/CRC/NRC's [16, 22]core idea is that test samples that are represented approximately by linear combinations of training samples from all classes, and then, the test samples are assigned to the corresponding class with minimum distance or approximate error. However, the coding coefficients in the sparse representation classifier SRC/CRC will be negative, which in practice makes the problem of the corresponding weights of positive and negative coding coefficients offset, which affects the sample classification accuracy to some extent. Nonnegative representation of classification NRC coding coefficients for classification ideas are restricted to nonnegative, and non-negative representation enhances the representation of homogeneous samples' capabilities while limiting the representation of heterogeneous samples. Despite the success of the three classifiers, SRC/CRC/NRC, in the image recognition task, they have their corresponding localization. When using the entire training image to reconstruct the test image *y*, on the one hand, both SRC and CRC are generated in the coding coefficient vector deviation. The reason is that from a generative point of view, it is not physically feasible to reconstruct real-world images from training images with complex negative (minus) and positive (plus) coefficients. NRC constrains the coding coefficients to be nonnegative, but due to the lack of proper regularization, NRC classification is not flexible enough to deal with real-world problems. NSCR [23] combines the advantages of sparse, collaborative, and nonnegative representations to be physically more robust and generalizable than previous sparse, collaborative, and nonnegative representations.

The NSCR classifier can be reconstructed as a bivariate problem bounded by a linear equation and can be solved under the alternate direction [24] method (ADMM) of the multiplicative subframe. Each subproblem can be solved efficiently in closed form and can converge to a global optimum. Extensive experiments of NSCR on various visual classification datasets have verified the effectiveness of NSCR classifier, and NSCR classification is better than advanced classification algorithms such as SVM and SRC. Based on the above discussion, the NSCR algorithm for a given test sample and training sample matrix *X*, *X* consists of several classes of samples, where *X* = [*X*_1_, ⋯, *X*′_*k*_] ∈ *R*^*D*×*N*^; its algorithmic idea is shown in Table 1:

### 2.3. Evaluation Metrics

In this paper, the evaluation metrics [25] include accuracy, sensitivity, specificity, *F*-score value, and Matthews correlation coefficient (MCC), which are described as follows:

Accuracy, sensitivity, and specificity were calculated by true positive (TP), false positive (FP), true negative (TN), and false negative (FN). TP indicates a benign tumor was predicted correctly, FP indicates a malignant tumor was predicted incorrectly, TN indicates a malignant image was predicted correctly, and FN indicates that benign tumors were predicted incorrectly. They are calculated by the following formulae. The calculation formula is as follows:

(3) $$\begin{array}{c} \text{Accuracy}=\frac{\text{TP}+\text{TN}}{\text{TP}+\text{TN}+\text{FP}+\text{FN}}, \end{array}$$

(4) $$\begin{array}{c} \text{Specificity}=\frac{\text{TP}}{\text{TP}+\text{FN}}, \end{array}$$

(5) $$\begin{array}{c} \text{Specificity}=\frac{\text{TN}}{\text{TN}+\text{FP}}. \end{array}$$

The *F* value is a summed average of the percentages of completeness and accuracy. It is used as a trade-off between accuracy and recall. The calculation formula is as follows:

(6) $$\begin{array}{c} F=\frac{2\times \text{TP}}{2\times \text{TP}+\text{FP}+\text{FN}}. \end{array}$$

MCC is a more comprehensive evaluation metric that reflects the reliability of the algorithm. When the number of categories is different, the value of the measure considered to be balanced ranges from -1 to +1. The MCC takes the value of 1 when the prediction error is 0 for both FP and FN, which means that the classification is completely correct; when the prediction error is 0 for both TP and TN, the MCC takes the value of -1, which means that the classification is completely wrong. It is calculated as follows:

(7) $$\begin{array}{c} \text{MCC}=\frac{\text{TP}\times \text{TN}-\text{FP}\times \text{FN}}{\sqrt{\left(\text{TP}+\text{FP}\right)\left(\text{TP}+\text{FN}\right)\left(\text{TN}+\text{FP}\right)\left(\text{TN}+\text{FN}\right)}}. \end{array}$$

## 3. NSRC-Based DenseNet Model

Target the network degradation problem when training CT modal medical images using convolutional neural networks, high dimensionality, and data redundancy during feature extraction and other problems. This paper combines the DenseNet-based feature extraction method and the classification recognition method based on nonnegative, sparse, and collaborative representation, in the proposal of a DenseNet-based nonnegative, sparse, and collaborative representation (DenseNet-NSCR) classification of benign and malignant lung tumors. The steps of the calculation as a whole are divided into image preprocessing, DenseNet feature extraction, and NSCR classification.

### 3.1. Image Preprocessing

(1) *Data collection*: 5000 raw images of lung CT models were collected from a hospital in Ningxia of China between 2014 and 2016. The number of both benign and malignant lung tumors was 2500 cases [26].

(2) *Data preprocessing*: the original images of the lung CT models have numbered accordingly and recolored into grayscale images. Based on the clinical markers, the focal areas were intercepted from the full-grayscale images and normalized to the same size as the ROI images, e.g., 64 px × 64 px, to obtain CT modal samples, which were divided into benign samples and lung malignancy samples. The benign sample and the lung malignancy sample were each 2500 samples. The two types of targets were divided into a test set and a training set of 4000 and 1000 cases, respectively, according to a certain ratio, and constructed with its corresponding binary labels, where the benign label is 1 and the lung malignancy label is 2.

### 3.2. Dense Neural Network-DenseNet

(1) *Transfer learning*: the dense neural network, DenseNet-201 model is first pretrained on a large natural image dataset, ImageNet, with the parameters from the pretrained network as the initialization parameters in the network where the growth rate of the DenseNet is *k* = 32 while the compression rate of the transition layer is *θ* = 0.5.

(2) *DenseNet partial feature extraction*: the datasets and labels are input into the pretrained dense neural network, DenseNet-201, respectively, and a single-module network based on the DenseNet model, which is CT-DenseNet, is constructed; DenseNet is trained to extract the feature vectors of training samples and test samples at the full-joint layer.

### 3.3. NSCR Classification Identification

Extract the feature vectors of training sample matrices and test sample matrices at the full connection layer of a DenseNet, input the feature matrix as an NSCR classifier, standardize all training sample matrices, and test sample matrices to the L_2 paradigm and solve the coefficient matrix, which in turn is used to find the reconstruction error for each category. Finally, the final classification identification is completed based on the similarity of the reconstruction residuals as follows:

1. For the training sample *X*′ = [*X*′_1_, ⋯, *X*′_*K*_], *X*_*i*_ ∈ CT, and for the testing sample *y*′ = [*y*′_1_.⋯, *y*′_*n*_], *y*_*i*_ ∈ CT. After dense neural network, DenseNet-201 feature extraction, a training sample matrix *X*′ = [*X*′_1_, ⋯, *X*′_*K*_], and a test sample matrix of the feature space *y*′ = [*y*′_1_.⋯, *y*′_*n*_] are obtained
2. Standardize each column *X*′ of the matrix and the query sample *y*′ to the range of L_2
3. The nonnegative sparse and collaborative representation processing of *y*′ with the training sample *X*′ in feature space is done to obtain the matrix of representation coefficient $\hat{c}$:

(8) $$\begin{array}{c} \hat{c}=\arg\;\min_{c}\left\|y^{\prime }=X^{\prime }c\right\|+\alpha {\left\|c\right\|}_{2}^{2}+\beta c\;s.t.c\geq 0 \end{array}$$

(4) Classify the residual similarity of nonnegative, sparse, and collaborative representation of test samples by training samples:

(9) $$\begin{array}{c} r_{k}={\left\|y^{\prime }-X\prime_{k}{\hat{c}}_{k}\right\|}_{2} \end{array}$$

(5) Output the label categories corresponding to the residual results:

(10) $$\begin{array}{c} \text{Label}\left(y^{\prime }\right)=\arg\;\min\left\{r_{k}\right\} \end{array}$$

**Figure 3:**
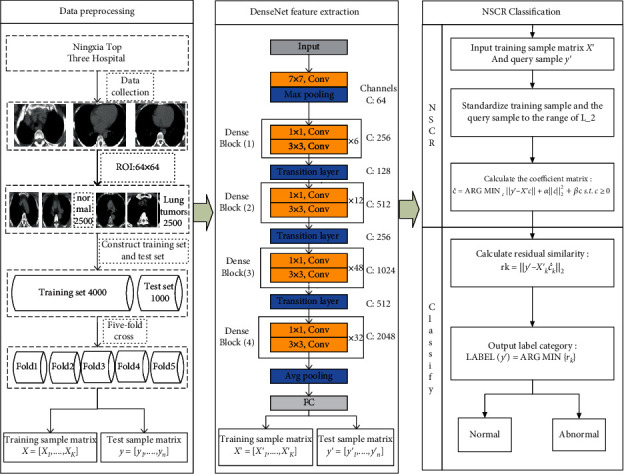
Flowchart of NRC DenseNet-based algorithm.

The NSCR-based DenseNet model DenseNet-NSCR is shown in Figure 3.

## 4. Algorithm Simulation Experiments

### 4.1. Experimental Environment

*Software environment*: Windows10 operating system, MatlabR2019a;

*Hardware environment*: Intel(R)Core(TM)i5-7200U CPU @2.50GHz 2.70GHz, 4.0GB memory, 500GB hard disk.

### 4.2. Results and Analysis of Experiments

To ensure the reliability of the data, the five-fold crossover method was used in this experiment. All samples were divided into five equal parts. Each copy contains equal proportions of the number of samples in different categories; 4 sets of data were used as training samples at a time, while the remaining 1 sample was used as a test sample, and each result was averaged to get the final result. That is, the number of training samples each session is 4000, the number of test samples is 1000, and the average of five experiments is taken. Experiments are conducted on three different network models, AlexNet, GoogleNet, and DenseNet, and three classification algorithms: the SVM, the SRC, and the NSCR. The results of the experimental comparison of the two combined models are as follows:

#### 4.2.1. Experiment 1: NSCR Regularization Parameter Optimization

The regularization parameters *α* and *β* affect the performance of the NSCR classifier to achieve the optimal performance of the NSCR classifier. In this experiment, the regularization parameters *α* and *β* were selected as 0.01, 0.05, 0.1, 0.5, and 1, respectively, with CT medical images as the dataset, the dataset was randomly divided 7 : 3, and a five-fold crossover experiment was performed. The optimal regularization parameters *α* and *β* were found with classification accuracy as the index.

**Figure 4:**
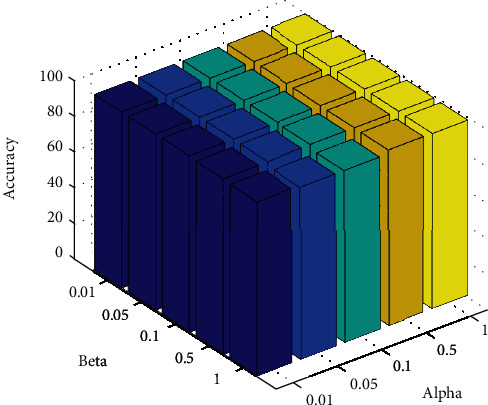
Regularization parameters *α* and *β*.

As shown in Table 2, the selection of different regularization parameters *α* and *β* affects the performance of the NSCR classifier. When *α* = 0.01 and *β* = 0.1, the NSCR classification accuracy is 99.48% and the performance of the NSCR classifier is optimal. To better indicate the effect of the selection of the regularization parameters *α* and *β* on the classification results, the three indicators were plotted in a three-dimensional histogram, as shown in Figure 4.

#### 4.2.2. Experiment 2: Comparison of Accuracy of Different Models and Time

This experiment focuses on the recognition accuracy and training time of nine algorithms (AlexNet+SVM, AlexNet+SRC, AlexNet+NSCR, GoogleNet+SVM, GoogleNet+SRC, GoogleNet+NSCR, DenseNet+SVM, DenseNet+SRC, and DenseNet+NSCR) for training and recognition on CT sampling space and probes the effects of different network models, different classification algorithms, and different sampling spaces on the recognition rate and training time of dense neural networks, as shown in Table 3.

In the first case, different network models are used with the same classification algorithm. In experiment 1, there are three sets of comparison experiments, namely, (AlexNet+SVM, GoogleNet+SVM, and AlexNet+SVM), (AlexNet+SRC, GoogleNet+SRC, and DenseNet-201+SRC), and (AlexNet+NSCR, GoogleNet+NSCR, and DenseNet-201+NSCR). To illustrate with the third group, in the CT sampling space, the accuracy of the DenseNet-201+NSCR model proposed in this paper is 0.28% and 0.58% higher, and the training time is 2460.66 s and 2900.24 s higher than the AlexNet+NSCR and GoogleNet+NSCR models, respectively. Not surprisingly, the DenseNet-201 has deep network layers, rich extracted image features, and high classification accuracy compared to other models. However, the cost is a significant increase in training time. The other two sets of results are similar and will not be recounted here.

In the second case, the same network and different classification algorithms are used. In experiment 1, there are three groups of comparison experiments, which are (AlexNet+SVM, AlexNet+SRC, and AlexNet+NSCR), (GoogleNet+SVM, GoogleNet+SRC, and GoogleNet+NSCR), and (DenseNet-201+SVM, DenseNet-201+SRC, and DenseNet-201+NSCR). To illustrate the third set, in the CT sample space, the classification accuracy of the DenseNet-201+NSCR model proposed in this paper is better than that of the DenseNet-201+SVM is 0.84% higher and 0.78% higher than DenseNet-201+SRC. In terms of training time, it is 51.97 s more than the DenseNet-201+SVM model and 727.69 s lower than the DenseNet-201+SRC model. Compared to the first two cases, the overall training time is significantly improved. However, after the network model is determined, the increase in training time complexity compared to the SVM classifier is relatively reduced. Moreover, the time complexity is significantly reduced compared to the SRC classification algorithm. Not surprisingly, under the same network model, the nonnegative, sparse, and collaborative representation classification algorithm NSCR has better classification accuracy, which better solves the optimization problem of high-dimensional data and with a much lower time cost compared to SVM and SRC.

#### 4.2.3. Experiment 3: Comparison of Different Combinations of Networks and Classifier Algorithms

The experiment focuses on nine algorithms (AlexNet+SVM, AlexNet+SRC, AlexNet+NSCR, GoogleNet+SVM, GoogleNet+SRC, GoogleNet+NSCR, DenseNet-201+SVM, DenseNet-201+SRC, and DenseNet-201+NSCR) trained on CT sampling space, in terms of accuracy, sensitivity, specificity, *F* value, and MCC for a total of five metrics to evaluate the merits of the algorithm. The results are shown in Table 4.

**Figure 5:**
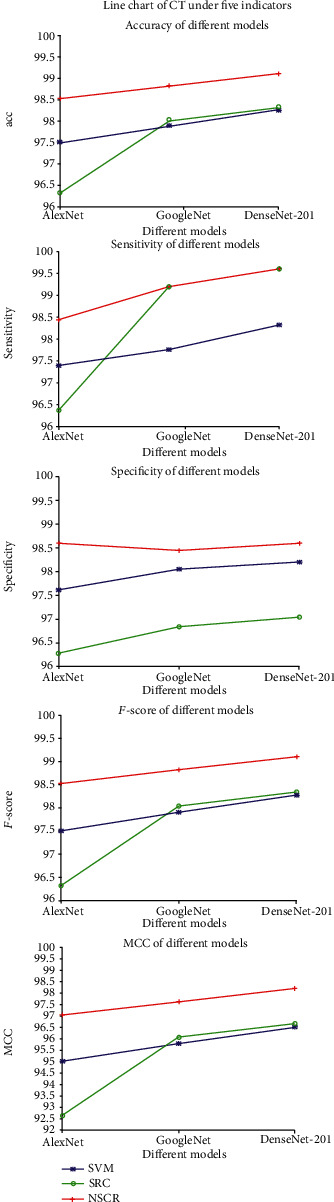
Line graphs of different indicators under CT and PET/CT datasets.

As shown in Table 4, the DenseNet-201+NSCR algorithm performance are all better than The DenseNet-201+NSCR algorithm has better metrics than other algorithms in terms of accuracy, sensitivity, specificity, *F* value, and MCC on the CT dataset improved by 2.78%, 3.24%, 2.32%, 2.78, and 5.56%, respectively. To point out the differences between the different algorithms on each indicator more clearly, the mean values of these five indicators are plotted on a line graph with the three network models in horizontal coordinates and the five evaluation indicators in vertical coordinates, respectively, as shown in Figure 5.

Through these two experiments and the correlation analysis, one can easily see that, with the same network model, this paper compares three classification algorithms, SVM, SRC, and NSCR, and the result of the experiments show that NSCR classification outperforms SVM and SRC classification algorithms for DenseNet in medical image extraction. The NSRC algorithm has better robustness for the problems of DenseNet in which features extracted from medical images appear to have high dimensionality and data redundancy. With the same classification algorithm, this paper compares three network models, AlexNet, GoogleNet, and DenseNet-201. The results show that DenseNet outperforms AlexNet and GoogleNet models, and DenseNet effectively uses high-level information to rediscover new features at the bottom layer, enhances the propagation of features across networks, and implements and strengthens feature reuse. The result shows that DenseNet outperforms AlexNet and GoogleNet, especially the DenseNet-201+NSCR model with deep network depth, strong network generalization capability, high classification accuracy, and better accuracy, sensitivity, specificity, *F* value, and MCC than the other models.

## 5. Conclusion

In this paper, a DenseNet based on nonnegative, sparse, and collaborative representation classification for benign and malignant classification of lung tumors (DenseNet-NSCR) is proposed. First, CT medical images were collected and preprocessed. The dataset is then trained in a DenseNet to construct a DenseNet model to extract the full connection layer feature vector. Finally, the lung tumor classification results were obtained in the NSCR classifier and compared by AlexNet+SVM, AlexNet+SRC, AlexNet+NSCR, GoogleNet+SVM, GoogleNet+SRC, GoogleNet+NSCR, DenseNet-201+SVM, DenseNet-201+SRC, and DenseNet-201+NSCR for a total of nine models. The DenseNet+NSCR model outperforms the other models with better robustness and generalization capabilities.

## Figures and Tables

**Table 1:** The NSCR-based classifier.

The NSCR based classifier	
1	Input: training sample matrix *X* = [*X*_1_, ⋯, *X*_*k*_] and query sample *y*
2	Normalize each column of matrix *X* and query sample *y* to the unit L_2 norm
3	The encoding vector of *y* on *X* is solved by the NSCR model
4	Calculate the coefficient matrix: $\hat{c}=\arg\;\min_{c}\left\|y^{\prime }=Xc\right\|+\alpha {\left\|c\right\|}_{2}^{2}+\beta c\;s.t.c\geq 0$
5	Calculate residual similarity: $r_{k}={\left\|y-X_{k}{\hat{c}}_{k}\right\|}_{2}$
6	Output label category: label(*y*) = arg min{*r*_*k*_}

**Table 2:** Accuracy under the regularization parameters.

	*α*					
*β*		0.01	0.05	0.1	0.5	1
	0.01	99.37	99.33	99.32	99.15	99.01
	0.05	99.47	99.36	99.33	99.17	99.01
	0.1	99.48	99.40	99.31	99.20	99.00
	0.5	99.03	99.15	99.24	99.12	98.93
	1	98.33	97.06	97.05	99.01	98.79

**Table 3:** Comparison of accuracy and training time results for different models.

Dataset	CT	
Model	Accuracy (/%)	Training time (/s)
AlexNet+SVM	97.50	224.58
AlexNet+SRC	96.32	604.20
AlexNet+NSCR	98.52	334.39
GoogleNet+SVM	97.90	662.44
GoogleNet+SRC	98.02	1081.21
GoogleNet+NSCR	98.82	773.97
DenseNet-201+SVM	98.26	3182.66
DenseNet-201+SRC	98.32	3962.32
DenseNet-201+NSCR	99.10	3234.63

**Table 4:** Comparison of CT results for different network models and classification algorithms.

Network model	Classification algorithm	Accuracy (%)	Sensitivity (%)	Specificity (%)	*F*-score (%)	MCC (%)
AlexNet	SVM	97.50	97.40	97.60	97.50	95.00
	SRC	96.32	96.36	96.28	96.32	92.64
	NSCR	98.52	98.44	98.60	98.52	97.04

GoogleNet	SVM	97.90	97.76	98.04	97.90	95.80
	SRC	98.02	99.20	96.84	98.04	96.07
	NSCR	98.82	99.20	98.44	98.82	97.64

DenseNet-201	SVM	98.26	98.32	98.20	98.26	96.52
	SRC	98.32	99.60	97.04	98.34	96.67
	NSCR	99.10	99.60	98.60	99.10	98.20

## Data Availability

The data used to support the findings of this study are available from the corresponding author upon request.
